# The Mechanism of Social Organization Participation in Natural Hazards Emergency Relief: A Case Study Based on the Social Network Analysis

**DOI:** 10.3390/ijerph16214110

**Published:** 2019-10-25

**Authors:** Yingxin Chen, Jing Zhang, Pandu R. Tadikamalla, Lei Zhou

**Affiliations:** 1 School of Economics and Management, Harbin Engineering University, Harbin 150001, China; chenyxdingdang@hrbeu.edu.cn (Y.C.); zhoulei960918@163.com (L.Z.); 2 School of Information Science and Engineering, University of Jinan, Jinan 250022, China; 3 Joseph M.Katz Graduate School of Business, University of Pittsburgh, Pittsburgh, PA 15260, USA; Pandu@katz.pitt.edu

**Keywords:** natural hazards, emergency relief, social network analysis, public participation, social organization, participation mechanism

## Abstract

The uncertainty and complexity of natural hazards put forward new requirements for emergency management systems. In order to deal with natural hazards effectively, it is important to build a cooperative network between government organizations and social organizations. The social network analysis method is adopted, the April 2013 Ya’an China earthquake is taken as a case study, the institutionalized emergency organization network before the disaster and the actual response organization network after the disaster are analyzed, and centrality, between centrality, closeness centrality and core-periphery are calculated. Through qualitative and quantitative research, the functions of social organization in the process of natural hazards emergency relief are revealed, the role orientation of social organization in the emergency management network is analyzed, and the influence factors of the social organization participation in the natural hazards relief is pointed out. Research results will help to promote the cooperation between social organization and government, and improve the efficiency of natural hazards emergency relief.

## 1. Introduction

In recent years, the frequent occurrence of natural hazards has caused great losses to people’s lives and property, and natural hazards always cascade to cause disasters [1]. For example, earthquakes often trigger a cascade of secondary disasters such as tsunamis, landslides, building and infrastructure collapses, etc. The disaster chain and cascading effect cause serious damage to buildings and large economic loss [2]. Facing the natural hazards, the emergency management system of the government usually falls into the bureaucratic dilemma. The low capacity of government and openness of information promote all kinds of social organizations to join the emergency management spontaneously, and bring resources such as information, materials, personnel and so on to the emergency management practice [3]. The ‘social organization’ adopted in this paper refers to the various forms of organizations that are spontaneously established by different social strata in the process of social transformation, to some extent, which are characterized by non-profit, non-governmental and social characteristics. Although the social organization is similar to the concept of Non-Profit Organization (NPO), Non-Governmental Organization (NGO) and ‘the third department’, it covers a wider range [4]. In the case of natural hazards, it is no use to distinguish among NPO, NGO and the third department. Therefore, the concept of social organization is more consistent with the actual situation of disaster response.

The participation of social organization in natural hazards emergency relief has two sides: On the one hand, the participation of social organization can make up for the functional defects of government. Social organization can make use of its own flexibility advantages to change functions and roles in time, get rid of the bureaucratic dilemma of government institutions, and improve the perception of the real environment and the adaptability to complex situations [5]. On the other hand, it increases the scale and heterogeneity of the actual response network after the disaster, thereby increasing the workload of emergency response coordination, and affecting the efficiency of emergency response coordination [6]. Therefore, how to make use of the information and resources of social organization, and promote the efficiency of emergency coordination between social organization and government has become an important issue in the field of emergency management.

This paper adopts the social network analysis method, through the empirical case study on the April 2013 Ya’an China earthquake, analysis the mechanism of social organization participation in natural hazards emergency relief, we specific analysis the multi-organization cooperation network, social organization’s function and role, and the influence factors of social organization function. Our research results provide theoretical support for promoting the collaboration between social organization and government. Specifically, we try to respond to the following questions:

What are institutionalized emergency organization network and the actual response organization network?
What’s the difference between the two networks?
What are the functions of social organization in natural hazards emergency relief?
What are the influencing factors of social organization function?

This paper will address these questions and take Ya’an earthquake as a case study. The remainder of this paper is structured as follows: In Section 2, we review the relevant literature and point out our contribution. In Section 3, we carry on case study of the Ya’an earthquake. Case description, data collection and processing workflow are presented in this section. In Section 4, we analyze case study results, the institutionalized emergency organization network and the actual response organization network are built. We compare the difference between two networks, and analyze the role of the social organization. In Section 5, we discuss case study results, point out the corresponding relationship between social organizations and their functions, and make clear the factors that affect the function of social organization. Final conclusions and an outlook on future search direction are given in Section 6. 

## 2. Literature Review

The research on social organization participation in natural hazards emergency management rose in the late 1990s [7]. On the one hand, the theories of government failure, civil society and multi-center governance have laid a theoretical foundation for the cooperation of the social organization and the government. On the other hand, the advantages of the social organization can make up for the shortcomings of the government in dealing with natural hazards, and provide the possibility for the cooperation [8]. Global scholars’ research mainly focuses on three aspects: (a) Participation pattern. (b) Societal, social and community resilience. (c) Participation model.

(1) Participation pattern

After the 2005 Hurricane Katrina, the participation of social organization in public crisis management and interdepartmental collaboration has been paid more and more attention [9]. Participation pattern change from top-down mode to bottom-up [10] and community-based model [11]. Skarbek (2014) adopted a novel set of comprehensive donation and expenditure data collected from archival records to examine a bottom-up relief effort in the Chicago Fire of 1871, result show that individuals, businesses, corporate entities and municipal governments are able to finance the relief though donations [12]. Thaler, et al. employed a mixed-methods approach, combining stakeholder workshops with a survey of 216 citizens at risk. Results show that bottom-up citizen initiatives can provide multiple benefits, such as increasing risk awareness and local adaptive capacities [13]. Ali, et al. encouraged full participation of government, private and public organizations. To institutionalize this effort, disaster organization based on the local conditions has been developed, increasing public knowledge and awareness [14]. Forino, et al. pointed out that local communities often enact Community-Based Initiatives (CBIs) to respond to climate change through Climate Change Adaptation (CCA). Partnerships should be established both among CBIs and between CBIs and City Councils and more communication needed among CBIs, City Councils and business actors [15]. Naim analyzed the interactive relationship between the government and the public from the micro-meso-macro perspectivesf, delineation of the boundaries between organizations, and facilitate better organization of cooperation [16]. 

According to the five core dimensions of cross-sectoral collaboration, Simo et al. affirmed the core role of regional NPO in the aspects of volunteer management, crisis environment adaptation, internal coordination and external coordination [17]. Caruson et al. put forward the establishment of regional emergency management cooperation organization at the local level, and promote the cooperation between government and social organization in information sharing, disaster preparedness, response and recovery [18]. Boin pointed out social forces such as enterprises, citizens and organizations should take an active part in dealing with public emergencies, and design cross-border, cross-departmental and cross-region emergency management system [19]. Based on the deviation between the actual response network and the national emergency plan, Kapucu et al. discussed the realistic structure of the collaboration system of government, private and social organizations [20]. 

Based on the case and qualitative method, Cent et al. analyzed the way and degree of the public participation in environmental protection according to the characteristics of the participants [21]. Sinuany-Stern et al. focused on the objective of improving operations and achievements of social organization, pointed out how to make use of available funds effectively to meet the demands and need for their services [22]. Gao pointed out that one important path to the development of new NGOs in Sichuan Province of China was a massive transfer of resources and personnel from other provincial governments. Pairing richer provinces with poorer ones to spur development is a PRC practice and is implemented when the earthquake struck [23].

(2) Societal, social and community resilience to disasters

Many scholars have studied societal, social and community resilience to disasters [24] Mamula-Seadon, et al. focused on some of the fundamental principles of sustainable risk management and societal resilience, proposed the mechanisms for integration and empowerment of local communities is essential for effective recovery and resilience [25]. Khalili, et al. identified the most essential social resilience indicators, and assessed these indicators through interviews with experts within the NSW State Emergency Service in regards to flooding of two case studies, and provided a framework for social resilience in different disaster phases [26]. Chiang, et al. highlighted the balance among built-environmental sensitivity and human adaptability dimensions, thereby emphasizing social vulnerability and addressing social resilience from risk perception perspectives [27]. Garcia, et al. analyzed the digital traces of 62,114 Twitter users after the Paris terrorist attacks of November 2015, and drew the conclusion that collective emotions after a disaster are associated with higher solidarity, revealing the social resilience of a community [28]. 

Kruse, et al. presented a framework of community resilience, which is a heuristic analytical tool for understanding, explaining and measuring community resilience to natural hazards. The framework conceptualizes resilience across three core domains: resources and capacities, actions and learning [29]. Kim, et al. explore climate justice and flood risk with specific reference to community resilience at the county-level, results suggest that community social and ecological characteristics were influenced by flood losses and that social capital and local proactive planning and policy measures lead to lower disaster losses and enhanced community resilience [30]. Ludin, et al. made use of the Index of Perceived Community Resilience (IPCR) and Buckner’s Index of Cohesion (BIC) to survey 386 flood evacuees from six communities in Kelantan, Malaysia, and their results showed that the higher social cohesion and social resilience, the higher disaster resilience [31].

(3) Participation model

Global scholars have carried out quantitative research and built many participation models. In order to assess social vulnerability (SV) to earthquake hazards. Zebardast presented the development of a hybrid factor analysis and analytic network process model for aggregating vulnerability indicators into a composite index of social vulnerability to earthquake hazards. The proposed model is then applied in Iran as a case study [32]. Werner analyzed the influencing factors of enterprise participation in public decision-making from three dimensions of reputation, politics and finance based on panel data [33]. Malone, et al. adopted a social networks analysis method and took a case study from Queensland, Australia, research on organizations involved in water management and flood responses events showed that cultural values were important in influencing network connections and preferred approaches to flood pre-planning and response [34]. Hogg, et al. analyzed the characteristics of 260 cross-sector community health networks that collectively consisted of 7816 organizations during the period 2008-15, and constructed community networks that include nonprofit, public, and private organizations [35]. 

Urrea, et al. made use of social networks to explore how structural factors affect humanitarian organizations’ performance in relief and development operations. Analyses of two recent humanitarian disasters show that having pre-established partnerships among implementers, a central coordinator, high connectivity, and few structural holes facilitates coordination and improves performance [36]. Mukhtarov, et al. pointed out that public participation is a central topic in urban water governance. They collected 33 published texts and discerned 32 case studies, which they analyzed according to the Cochrane systematic review methodology and found that Information and Communication Technologies (ICT) tools allow many citizens to be better informed and co-produce water services with a government [37]. Moroto, et al. elucidated the spatial distribution of NGOs working in disaster management in Bangladesh for the past seven years with a geographic information system (GIS) and used logistic regression to analyze possible factors influencing their decisions on project locations, and revealed the socially disaster vulnerable areas where NGOs are less likely to intervene [38]. Simsa, et al. analyzed the experiences of spontaneous volunteers (SVs) working under the auspices of civil society organizations and derived management implications. The results show that the environment of spontaneous volunteering in social crises differs from that in natural hazards situations. SVs partly substitute official response systems and this lead to a high degree of self-organization [39]. 

To sum up, many scholars have studied social organization participation in emergency management from different perspectives, and mainly agree with the following points: First, the collaborative governance of emergency management is very important. Civil forces, enterprises, government and NGO should actively participate in natural hazards response. Second, social organizations have gradually stepped onto the historical stage and played an important role in emergency management. 

At present, the social network analysis is good method used to study the cooperative network structure in emergency management [40]. At the same time, foreign research on the cooperation network in emergency management is based on the unique government system of various countries, which is not suitable for China’s national conditions, which provides space for our research and highlights the theoretical and practical value of this paper.

The contributions of this research are as follows:

Taking the Ya’an earthquake as the research sample, based on social network analysis method, the institutionalized emergency organization network and actual response organization network are constructed, and two kinds of network structure are compared. Research results are helpful to effective evaluate social organization’s functions in the natural hazards emergency relief, and provide reasonable assistance for emergency management.
We make clear the function and role of social organization in the process of natural hazards emergency relief, classify social organization and point out its corresponding function. Research results will help to optimize the emergency management network structure. Studying the participation form and network status of social organization can help to promote the cooperation between social organization and government, and improve the adaptability of emergency system.

The adoption of empirical investigation and social network analysis method has promoted the expansion of research focus from theoretical discussion to empirical research, and from qualitative analysis to quantitative analysis, so as to further improve the theory of emergency management. Natural hazards management in China is a ‘command-control’ hierarchical structure, which is difficult to meet rescue needs. A social organizations cooperative network is proposed in this paper, which contributes to the transformation and development of traditional organizational theory.

## 3. Case Study of Ya’an Earthquake

### 3.1. Case Description

**Figure 1:**
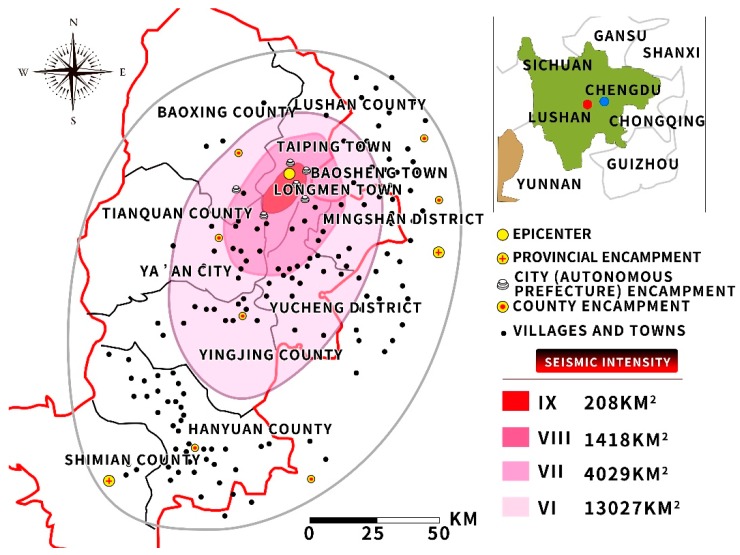
Study area map.

In April 20, 2013, a 7 Ms earthquake occurred in Lushan County (Ya’an, Sichuan Province, China). The depth of the earthquake was 13 km, the epicentral intensity was 6.4 degrees, and the aftershock was 8182 times. A total of 193 people were killed, 25 were missing, and the number of victims was 383 thousand. Only at the epicentre in Lushan County, the direct economic loss was up to 85 billion 171 million yuan [41]. The study area map is given in Figure 1.

There are two reasons for choosing the Ya’an earthquake as the case study. First of all, the Ya’an earthquake was 7 Ms, which is devastating and representative. Secondly, after the Wenchuan earthquake in 2008, Sichuan Province has accumulated abundant experience in earthquake relief, and released a more perfect earthquake emergency plan, so the Ya’an earthquake can serve to verify the effectiveness of the updated emergency plan, and provide a good case for the research of cooperative network structure.

### 3.2. Data Collection and Processing

**Figure 2:**
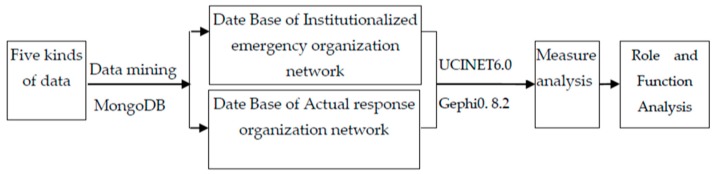
The workflow of case study.

In this study, a quantitative research method is adopted, guided by trans-department collaboration theory. In the early stage of the outbreak of earthquake, the MongoDB database is used to track relevant information, and multi-channel information collection mode is applied to break through single sample restriction. We mainly collect five kinds of data. The first is documents about earthquake emergency plans issued by the government website before April 20, 2013. The data range is the earthquake emergency plan of national government, provincial government and municipal government, and laws and regulations on the official websites. The second types of data are mainly from the official websites of the China Earthquake Administration, the Sichuan earthquake bureau, the Sichuan provincial government, the Ya’an municipal government and so on. The third type of data collects from the NGOs’ official websites such as One Foundation, China Charity Federation, Red Cross Society of China, Red Cross of Chengdu and so on. The fourth kind of data collects from the traditional news portal, such as Xinhua website, Renmin website, Phoenix website and so on. The fifth type of data sources are Sina, Tencent and Sohu and other micro-blog new media platforms. The data source not only ensures the comprehensiveness and effectiveness, but also takes into account the differences between the traditional media and the new media. We track the relevant data in a month (from April 20, 2013 to May 20, 2013) after the earthquake, and all data are inputted into UCINET6.0 and Gephi0. 8.2 [42] to be analyzed. The workflow of case study is given in Figure 2.

## 4. Result Analysis

### 4.1. Institutionalized Emergency Organization Network

The institutionalized network of emergency organization is a network formed by various cooperation organizations according to the emergency plan before natural hazards occurrence. In reference to the ’The composition of the working group of the General Command Department of earthquake relief’ published by the state council in May 18, 2008, the earthquake relief organization is divided into nine working groups: rescue and relief group, mass life group, earthquake monitoring group, epidemic prevention group, propaganda group, production recovery group, infrastructure protection and reconstruction group, water conservancy group and social security group [43].

**Figure 3:**
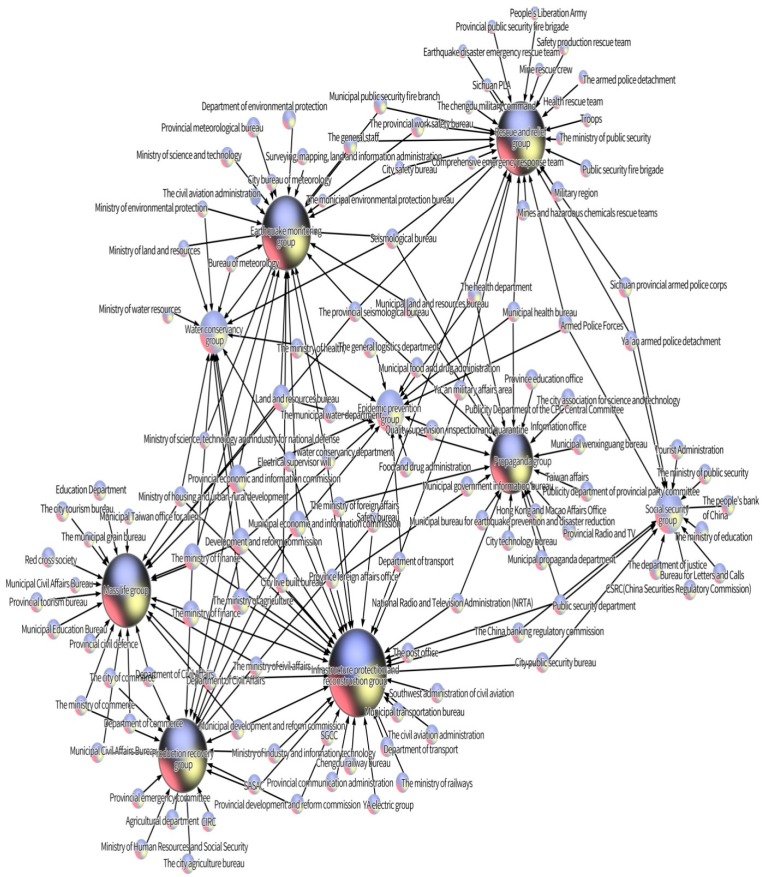
Institutionalized emergency organization network (due to limited space, this figure is blurry; readers may e-mail the authors for a clearer figure).

According to the document made up by the Ya’an earthquake relief working group, 81 organizations have been identified to be involved in the earthquake relief. In addition, from three angles of ‘state-provinces-city’, we retrieve keywords: ’national earthquake emergency plan’, ‘Sichuan province earthquake emergency plan’ and ‘Ya’an earthquake emergency plan’ on official websites of the state council, the Sichuan seismological bureau and Ya’an city, and distinguish nine, 59 and 65 cooperation organizations respectively. We eliminate repetition organizations and summarize a total of 122 organizations which constitute institutionalized emergency organization network as shown in Figure 3.

### 4.2. Actual Response Organization Network

**Figure 4:**
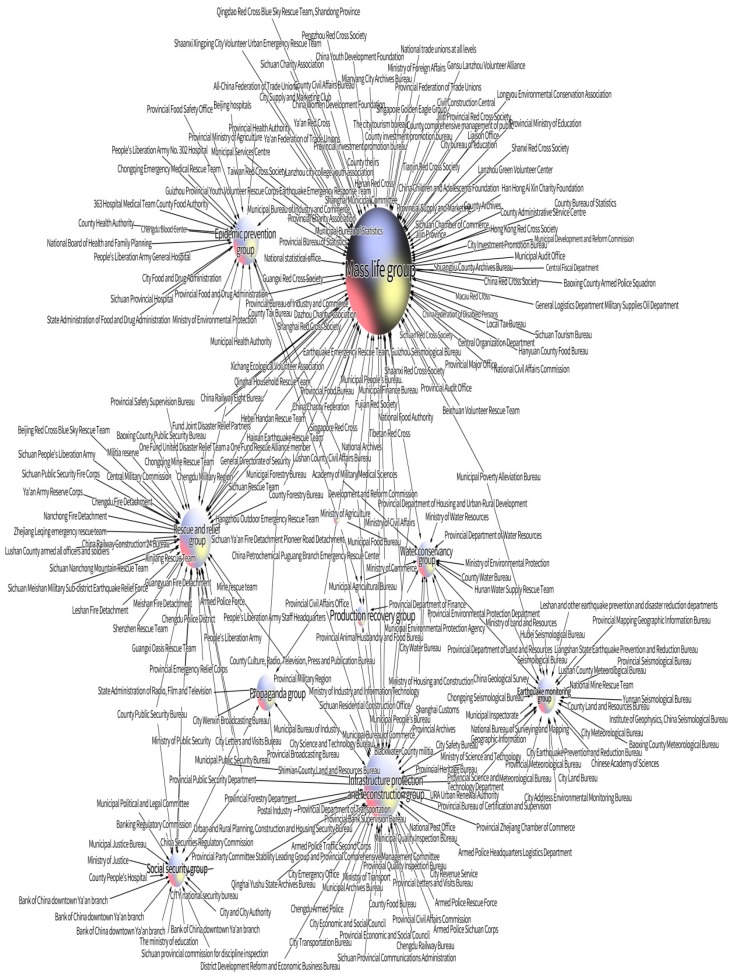
Actual response organization network (due to limited space, the figure is blurry; readers may e-mail the authors for a clearer figure).

The actual response organization network is the cooperation network that many organizations participate in the emergency relief after the earthquake, namely the cooperation network composed by the government organizations and the social organizations. According to the data sources mentioned in Section 3.2, 262 cooperative organizations are identified in the actual response organization network as shown in Figure 4.

By comparing Figure 3 and Figure 4, we find that the actual response organization network has been significantly increased in network size and heterogeneity. 262 social organizations and 1447 organization interaction are identified in actual response organization network. From Figure 4, we can see that 105 organizations participation in the ‘mass life group’, including 51 government organizations and 54 social organizations. Many NGOs have participated in this group, such as Red Cross of Shanghai, Red Cross of Shanxi and Red Cross of Singapore, Xichang Ecological Volunteer Association, Lanzhou Green Space Volunteer Center, Lanzhou City University Youth Association, Hanhong Love Charity Federation, China Charity Federation and so on. The two social organizations, One Foundation Disaster Relief and One Foundation Rescue Alliance, consist of 47 and 18 civil relief organizations respectively, it is show that the non-government power is strong. There are 58 organizations and 47 organizations involved in the ‘infrastructure protection and reconstruction group’ and the ‘rescue and relief group’ respectively. There are only 6 organizations involved in the ‘production recovery group’. The cross-border interaction between different organizations is shown in Table 1. 

The total cross-border interaction is 427, accounting for 29.5% of the interaction of all organizations. The cross-border interaction centered on NGOs and state owned enterprises is 102, accounting for 7% of the interaction of all organizations. This shows that more interaction occurs mainly among similar organizations, namely interaction always between public organizations and public organizations, state-owned enterprises and state-owned enterprises, public institutions and public institutions, private enterprises and private enterprises, NGO and NGO.

### 4.3. The Structure and Role Analysis of Actual Response Organization Network 

#### 4.3.1. Network Density

The network density is a measure of interconnection degree among the nodes in the network, the value range generally is in [0,1] [44]. For a valued-directed network, its network density may exceed 1 because its relation matrix does not belong to the Boolean matrix. In the directed network including *n* nodes, the maximum continuous number is *n* (*n* − 1). The network density is given in Equation (1):

(1) $$D=\sum l_{w}/n(n-1)$$

where, $\sum l_{w}$ is the sum of all edges values in the valued-directed graph. The measurement results of the network density of the Ya’an earthquake is given in Table 2.

From Table 2 we can see that the network density of valued-directed network is 3.15, while converting the relation matrix into a Boolean matrix, the network density is only 0.41. Therefore, the network density has a direct correlation with the strength of the relationship between nodes, and the network density value will decrease with the increase of the number of nodes.

#### 4.3.2. Centrality

Centrality is the structural feature of the individual node in the network. The greater the node’s centrality, the greater the reputation, status, power and importance of node in the network, and the stronger ability to acquire information [45]. The degree centrality is given in Equation (2), which represent the ability of the node to interact directly with other nodes in a network, the greater the *CD*(*i*) is, the stronger the node’s ability to interact with other nodes, the more likely the node is to become the center of the network, and the greater the power it has. The relative centrality is given in Equation (3):

(2) $$C_{D}(i)\;=d(i)=\sum_{j}X_{ij}=\sum_{i}X_{ji}$$

(3) $$C_{DS}(i)=C_{D}(i)/g-1=d(i)/g-1$$

where *C_D_*(*i*) is absolute centrality, *d*(*i*) is the number of other nodes that node *i* connection, *i* and *j* represent different organizations, if there is a direct connection between *i* and *j*, then *X_ij_* = 1, otherwise *X_ij_* = 0. *C_DS_*(*i*) is relative centrality, *g* represents the number of nodes in the network.

#### 4.3.3. Between Centrality

In a network with *n* nodes, the between centrality is the degree of the node control the association with other nodes [46]. The absolute between centrality is given in Equation (4), where *C_ABi_* is between centrality, *T_jk_* represents the number of shortcuts between nodes *j* and *K, P_jk_*(*i*) represents the probability of node *i* on the shortcut between nodes *j* and *K,* and *P_jk_*(*i*)* = T_jk_*(*i*)*/T_jk_*. In order to compare the between centrality of the different networks, it is standardized to relative between centrality *C_RBi_*, and the expression is given in Equation (5). The value range of relative between centrality is in [0,1]. If the relative between centrality of the node is 0, it indicates that the node does not control any other nodes and is at the edge of the entire network. If the relative between centrality of the node is 1, it indicates that the node can control any other nodes and is in the center of the whole network, and has higher rights and prestige:

(4) $$C_{\mathrm{ABi}}=\sum_{j}^{n}\sum_{k}^{n}P_{jk}(i)\;j\neq j\neq i,j<k$$

*C_RBi_* = 2*C_ABi_*/(*n*^2^ − 3*n* + 2) (5)

#### 4.3.4. Closeness Centrality

In the network with *n* nodes, the closeness centrality is the proximity degree between node i and other nodes [46]. The relative closeness centrality is given in formula (6), where *di*(*ci*,*cj*) represents the number of shortcuts that connect nodes *i* and *j*, the value range of relative closeness centrality is in (0,1]. If the relative closeness centrality is 0, it indicates that the node is at the core and not controlled by other nodes. If the relative closeness centrality is 1, it shows that the node is at the edge and is controlled by other nodes:

(6) $$C_{c}(c_{i})=(n-1)/\sum_{j=1}^{n}d_{i}(c_{i},c_{j})$$

The measure values of the actual response organization network are given in Table 3.

If the node has bigger degree centrality and the between centrality, and smaller closeness centrality, then the node has higher network centrality. As can be seen from Table 3, the top five nodes are the People’s Government of Sichuan Province, Provincial Earthquake Relief Command Department, Provincial Disaster Relief Material Reserve, Ya’an Municipal People’s Government and Sichuan Provincial Committee of the Communist Youth League. They have higher network centrality (the degree centrality and the between centrality are bigger, and the closeness centrality is smaller). Most of the top ten nodes are government organizations. Among them, the degree centrality and the between centrality of the People’s Government of Sichuan Province are 0.846 and 0.087 respectively, which are biggest, and closeness centrality is 0.409 which is smallest. It shows that the People’s Government of Sichuan Province’s network centrality is the strongest and plays an important role in the whole network. It has the most direct interaction with other nodes, and has strong ability to control resources. Among social organizations, the degree centrality and the between degree of China Foundation 4.20 Self-discipline Alliance are 0.387 and 0.068, respectively, which are greater than that of other social organizations. It shows that compared with other social organizations, China Foundation 4.20 Self-discipline Alliance has strong resource control advantages. On the one hand, it integrates many kinds of foundation organizations such as One Foundation, China Youth Development Foundation, China Soong Ching Ling Foundation, Alibaba Foundation and so on. On the other hand, it links with government organizations, and acts as a bridge between government and social organizations in cooperative networks.

#### 4.3.5. Core—Periphery

The core-periphery theory is applied to the social network analysis. Nodes and nodes are interconnected, forming the core area by closely linked nodes and the marginal zone by sparse linked nodes [47]. The core-periphery value of the actual response organization network is given in Table 4.

From Table 4 we can see that the core degree and periphery degree are 0.309 and 0.062 respectively, there are significant differences between them, indicating the existence of core- periphery relationship. The core areas include ‘mass life group’, ‘rescue and relief group’, and ‘infrastructure protection and reconstruction group’, and remaining 6 working groups belonging to the marginal zone. The organizations in the core area are government organizations such as the Provincial Civil Affairs Department, the Provincial Public Security Department, and the Ministry of Civil Affairs, only a few social organizations such as the Red Cross Society of Shanxi and Red Cross Society of Shanxi are at the core area. This shows that in the cooperation network, the government organization is still in the dominate position and has plenty of resources, and the rescue force of the social organizations make up for the shortage of the government.

## 5. Result Discussion

### 5.1. The Function of the Social Organization

**Figure 5:**
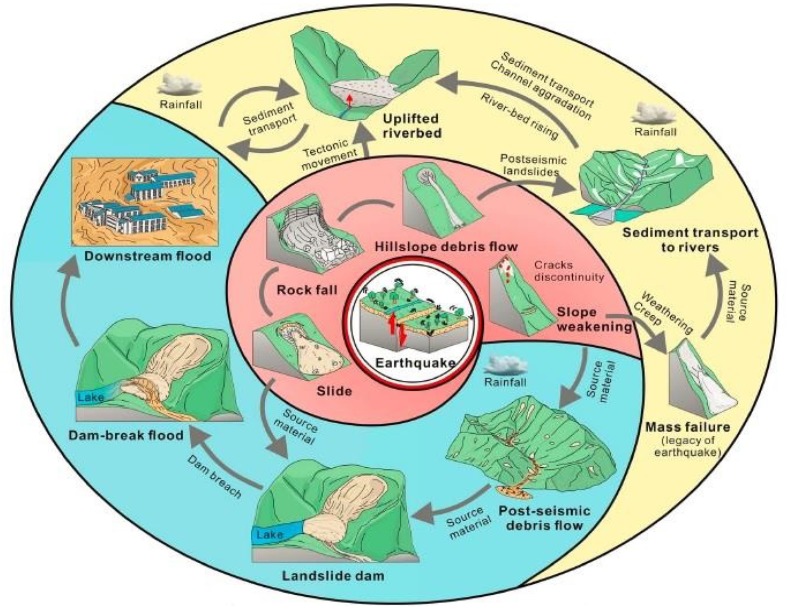
Geologic cascading hazards triggered by earthquake.

Natural hazards such as earthquake can cause a series of cascading hazards, as shown in Figure 5.

**Figure 6:**
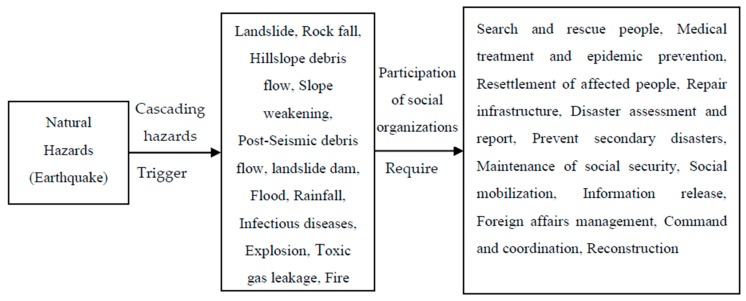
The relation between cascading hazards and social organizations’ participation.

According to the Emergency Support Function released by Federal Emergency Management Agency and the Earthquake Emergency Plan issued by China government in August 28, 2012, we set up a function classification for the organizations, including the following 12 items: (1) Search and rescue people (2) Medical treatment and epidemic prevention (3) Resettlement of affected people (4) Repair infrastructure (5) Disaster assessment and report (6) Prevent secondary disasters (7) Maintenance of social security (8) Social mobilization (9)Information release (10) Foreign affairs management (11) Command and coordination (12) Reconstruction. Social organizations’ participation in natural hazards emergency relief includes the whole process of cascading hazards. The relation between cascading hazards and social organizations’ participation is given in Figure 6.

Disaster risk reduction strategies and functions of social organizations in this framework are given in Table 5.

The classification and function of the actual response organization network is given in Table 6.

**Figure 7:**
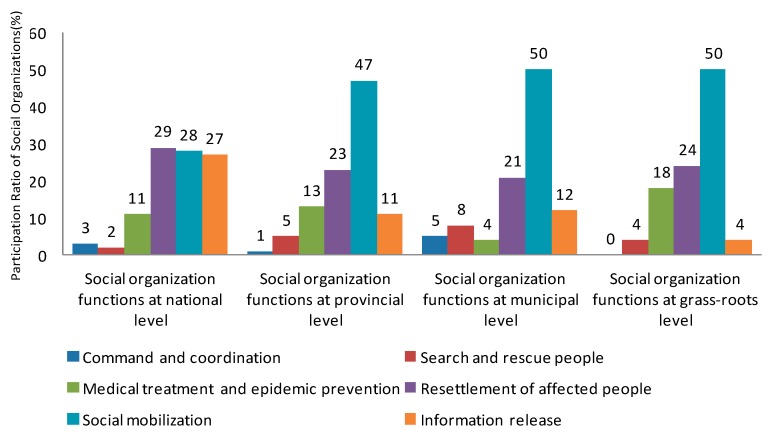
Main functions of social organization at different levels.

In the process of emergency relief for natural hazards, the most important functions of social organizations are command and coordination, search and rescue people, medical treatment and epidemic prevention, resettlement of affected people, social mobilization and information release. The main functions of social organizations are analyzed from four different levels: state, province, city and grass-roots level, as shown in Figure 7.

The social organization participating in the rescue and relief are usually spindle shape distributed in the provincial and municipal levels. The first reason is that the management registration system is restricted, and the organization tends to find Chief of Operations at the municipal level, and to obtain favorable resources. The second reason is that the development of grassroots social organizations is not yet perfect, the public welfare organizations based on the community needs further development and improvement. This shows that power transfer from government to social organization is still in the primary stage, and a powerful civil society needs to be formed.

From Figure 7, we can see that the functions of social organizations mainly include the social mobilization and the resettlement of the affected people, but their focus is different. Depending on medical, material, human resources and communication mechanism, national social organizations, including official social organizations and social service organizations, can gain the trust of the government, so the national social organizations are outstanding in command, coordination and information release. The low level organizations do not have the advantage of government trust, and lack of independence and the right to speak, so they can only play the function of charitable mobilization. Thus, the official social organizations and social service organizations have resource that government given, they squeeze NGOs’ activities range. It is noteworthy that the performance of professional social organizations are not prominent, the social organization needs to be further developed.

Because the centralization politics in China is different from federalism in other countries, we analyze the similarities and differences of the social organizations participation in natural hazards emergency relief in a different political context:

(1) In China’s centralized political situation, the official social organizations account for the largest proportion of the actual response organization network. In the actual response organization network of the Ya’an earthquake, the proportion of official social organizations, social service organizations, civil society organizations and enterprise social organizations are 40.2%, 30.3%, 20.6%, and 8.9% respectively. This result is different from that in the federalist political system. For example, the actual response network of September 11 attacks analyzed by Comfort include 44.6% of official social organizations, 16.9% of civil society organizations and 35.8% of private enterprise [48].The actual response network of Hurricane Katrina include 57% of official social organizations, 15.7% of NGO and 26.7% private enterprise [49]. In these two political environments, the main body of the actual response network is the government, but in China’s political environment, official social organizations account for a larger proportion, while in the federal political environment, enterprise social organizations account for a larger proportion.
(2) The scale and heterogeneity of the actual response organization network greatly increased compared with the institutionalized emergency organization network. This result is consistent with the research conclusion in the federalist political situation [50]. In China’s centralized political situation, functions of social organization include search and rescue people, medical treatment and epidemic prevention, resettlement of affected people, repair infrastructure, disaster assessment and report, prevent secondary disasters, maintenance of social security, social mobilization, information release, foreign affairs management, command and coordination, and reconstruction. Among them, the function of social mobilization is the most remarkable, while the functions of social security maintenance are the least remarkable. These functions are generally similar to those of social organizations in the federal political environment [51].

### 5.2. The Influence Factors of the Social Organization Function

In Chinese centralized political situation, the three dimensions: social value, social relation and resource ability affect the function of the social organization. First of all, in social value dimension, China has been taking more stringent restrictions on the development of social organization for the past years, so the legal development space of social organization is very limited. However, due to the disintegration of the ’unit system’ and the public is reduced to ’atomized’ individuals. It is difficult for the government to directly communicate with billions of atomized individuals. Therefore, the actual situation is that legally registered social organizations in China are not so much, but there are many social organizations which actual existence without registration and very active. After the Wenchuan earthquake in 2008, Chinese social organizations began to participate in natural hazards emergency relief. It became a complement to the institutionalized emergency response network and got trust and support of the Chinese government. Some of the social organizations gained independent legal identity. For example, the One Foundation, which was originally affiliated with the Red Cross Society of China, could not raise money independently. In 2011, to a large extent, due to the good performance in the Wenchuan earthquake in 2008, the One Foundation was registered in Shenzhen and acquired legal status. Therefore, in the Chinese political situation, social organization’s participation in disaster relief is not only by the moral drive, but also from the motivation of proving its own legitimacy. Driven by this dual power, social organizations participation in natural hazards emergency relief becomes a common practice.

Secondly, in the social relation dimension, in Chinese tripartite relationship of government, market and society, it is apparent that the government is the strongest, followed by the market and the society is the weakest; but in fact, it is not the case. If the state-owned enterprises are excluded from the market, and the NGOs and the volunteers can be included in the society organizations, then the ternary relationship among the government, market and society may need to be restated: the government is the strongest, the society is the second, and the market is the weakest. This is consistent with the Chinese reality, state-owned enterprise leaders are appointed by the government, is the extension of the government function, especially after nearly ten years of ’the state advances and the private sector retreats’, the proportion of the state-owned enterprises in the market economy is further increased, and the private enterprises are shrinking. In the actual response network after the Ya’an earthquake, once the state-owned enterprises are listed as a separate category, the proportion of private enterprises is very small. 

Finally, in the resource ability dimension, although the number of social organizations involved in the Ya’an earthquake is large, the resources and capacity are still insufficient. Most of social organizations are small, lack of continuous source, logistical support and information collection capabilities. It is difficult for them to perform their own tasks or run projects independently, and sometimes even need the government to allocate tasks. Private enterprises have only a few ways of participation, mainly through donations. The shortage of resources and low capacity also restricts the function of social organizations.

## 6. Conclusions

From 2008 to 2013, social organizations have made great progress, such as money raising, self- management, personnel scheduling, material allocation, organization coordination, information exchange and so on. Many types of social organizations at different levels, regions and areas are actively involved in natural hazards emergency relief, and under the guidance of core organizations, a collaboration network is initially formed. By making use of the advantages of flattening and flexibility, social organizations give full play to their functions and roles to make up for the defects of government bureaucracy, and improve the ability of emergency management system to perceive the real environment and adapt to complex situations. But in the real emergency response cases, most of the social organizations are still in a loose and dissociate state, and can’t occupy the core position in a cross sectoral collaboration network. 

In order to give full play to the function and role of the social organization, we put forward the following countermeasures and suggestions:

First of all, strengthen the legalization of social organizations. At present, the public calls for the government to accelerate the construction of laws and regulations for social organizations. The government should make clear the legal status of social organizations in the natural hazards emergency plan, so that social organizations can maintain their legitimate rights and interests.

Secondly, enhance communication and coordination among organizations. The Wenchuan earthquake and the Ya’an earthquake have strengthened the communication and cooperation among the government and NGO, but the communication among government organizations, NGO, NPO and other organizations is still less. The government can consider transfer part work from the ’mass life group’ to social organizations, because the social organizations have professional advantage. The social organizations are usually shortage of funds, the government should set up a corresponding support system.

Thirdly, local organizations at all levels should gain more rights. Local organizations are mostly originating in the lower level and can understand and meet the needs of the people as soon as possible. Centralization has a tendency to decentralization, and the approval mechanism has become more flexible and the decision-making factors will be reduced, which makes the local organizations respond to the disasters faster.

Finally, more attention must be paid to the cascading hazards. Natural hazards usually cause cascading hazards. Social organization participation in emergency relief in the whole process of cascading hazards can improve rescue efficiency, contribute to the work of disaster prevention and mitigation, so as to improve the credibility of the government and maintain social stability.

In the future, we will have two research directions. The first is a multi-case analysis, as only Ya’an earthquake is analyzed in this paper, but there are many different natural hazards, such as floods, hurricanes, tornadoes and so on. Is social organizations’ participation mechanism suitable for other natural hazards? The participation mechanism in different natural hazards needs to be further studied. The second is multi-organization cooperation mechanism, only cooperation network is studied in this paper. How to efficiently collaborate among multi-organizations is a further research direction.

## Figures and Tables

**Table 1:** Cross-border interaction between different types of organizations.

Organization Type	Public	State-Owned	Nonprofit	Private	Public Institutional
Public		65.0	39.0	35.0	32.0
State-owned	65.0		30.0	8.0	2.0
Nonprofit	39.0	30.0		65.0	26.0
Private	35.0	8.0	65.0		6.0
Public Institutional	32.0	2.0	26.0	6.0	

**Table 2:** Network density.

Network Density of Valued-Directed Network	Network Density of a Directed Boolean Matrix
Density (matrix average)=3.1547	Density (matrix average) = 0.4136
Standard deviation=6.9511	Standard deviation = 0.4928

**Table 3:** The measure values of the actual response organization network.

Degree Centrality (Top Ten)		Between Centrality (Top Ten)		Closeness Centrality (Top Ten)	
the People’s Government of Sichuan Province	0.846	the People’s Government of Sichuan Province	0.087	the People’s Government of Sichuan Province	0.409
Ya’an Municipal People’s Government	0.645	Provincial Earthquake Relief Command Department	0.085	Provincial Earthquake Relief Command Department	0.415
Emergency Material Distribution Center of Ya’an	0.556	Ya’an Municipal People’s Government	0.076	Provincial Disaster Relief Material Reserve	0.427
Provincial Disaster Relief Material Reserve	0.432	Provincial Disaster Relief Material Reserve	0.071	Ya’an Municipal People’s Government	0.438
Provincial Earthquake Relief Command Department	0.413	China Foundation 4.20 Self-discipline Alliance	0.068	Department of Civil Affairs of Sichuan Province	0.543
China Foundation 4.20 Self-discipline Alliance	0.387	Emergency Material Distribution Center of Ya’an	0.065	Sichuan Earthquake Administration	0.565
China Earthquake Administration	0.358	China Earthquake Administration	0.063	Sichuan Provincial Postal Administration	0.615
Sichuan Provincial Committee of the Communist Youth League	0.323	Sichuan Provincial Committee of the Communist Youth League	0.059	Sichuan Provincial Committee of the Communist Youth League	0.634
Department of Civil Affairs of Sichuan Province	0.296	Health and Family Planning Commission of Sichuan Province	0.052	Sichuan Provincial Finance Department	0.721
Red Cross Society of China	0.267	Sichuan Provincial Finance Department	0.048	Beijing Municipal Government	0.748

**Table 4:** Core-periphery value.

Density Matrix			Final Fitness
	1	2	0.332
1	0.309	0.072	
2	0.225	0.062	

**Table 5:** Disaster risk reduction strategies and corresponding social organizations’ functions.

Function	Disaster Risk Reduction Strategies
Search and rescue people	Grass-roots emergency teams and people’s self-help and mutual rescue
	Deploy rescue equipment to rescue the victims
	Communication and cooperation between rescue teams
Medical treatment and epidemic prevention	Emergency medical teams rushed to the scene to rescue victims
	Allocation of ambulances, medical devices, drugs and plasma
	Coordination and Distribution of medical resources in surrounding areas
	Psychological assistance
	Health and epidemic prevention in disaster areas
Resettlement of affected people	Open up emergency shelters, raise and transport all kinds of relief materials
	Set up distribution points for daily necessities. Produce, transport and install prefabricated houses.
	Provide necessary fire-fighting equipments
	Mobilize social forces to settle the affected people
	Do a good job in the aftermath of the victims and comfort their families
	Organize schools in disaster areas to resume classes
Repair infrastructure	Rush repair of airports, railways, highways, bridges, tunnels and other transport facilities
	Rush repair of power supply, water supply, gas supply, communications, radio and television and other infrastructure
Disaster assessment and report	Strengthen meteorological monitoring and pay close attention to major meteorological changes in disaster areas
	Strengthen monitoring of air, water and soil pollution
	Investigate the affected degree of disaster area and carry out disaster damage assessment
	Report the disaster damage assessment to the State Council
Prevent secondary disasters	Strengthen the monitoring and early warning of secondary disasters and prevent landslides, debris flows, rolling stones, etc.
	Carry out investigation, assessment and reinforcement of dangerous situations in reservoirs, hydropower stations, dams and barrier lakes, and organize personnel transfer from dangerous areas when necessary
	Strengthen the investigation of hazardous chemicals production and storage equipment, oil and gas pipelines, transmission and distribution lines, and take timely safety precautions
Maintenance of social security	Fighting against illegal and criminal acts such as theft, robbery, looting of materials and spreading rumors
	Additional temporary police stations shall be set up in areas such as mass resettlement sites and material storage sites.
	Resolve conflicts and disputes and provide legal services
Social mobilization	Disaster Relief headquarters Strengthen Volunteer Service Management
	Do a good job in volunteer dispatch and related services
	Publish the volunteer service requirements to the public and guide volunteers to participate safely and orderly
	Donation organization, receipt, statistics, distribution, use, public feedback, etc.
	Organize people’s governments in non-disaster areas to provide counterpart support to the people in disaster areas
Information release	Disaster relief headquarters at different levels are responsible for the release of disaster information at corresponding levels
Foreign affairs management	Inform relevant countries and regions of the disaster situation
	Coordinate and arrange the entry of foreign rescue teams
	Receive and manage overseas relief materials and arrange interviews with overseas media
Command and coordination	The General Staff Department and the Civil Aviation Administration exercise flight control and conduct aerial reconnaissance
	The Ministry of Industry and Information Technology coordinates communications resources to ensure smooth communications
	The disaster relief headquarters of the state council coordinate, organize, guide and dispatch disaster relief work
	Provincial disaster relief headquarters organizes professional disaster relief teams
	Municipal (prefectural) and county disaster relief headquarters organize grass-roots self-rescue and mutual rescue
Reconstruction	The State Council and provincial governments prepare post-disaster recovery and reconstruction plans
	Governments at all levels in disaster-stricken areas carry out restoration and reconstruction, and relevant departments of higher-level governments give support and guidance.

**Table 6:** The classification and functions of social organization.

Organization type	Constitute	Function
Official social organization	People’s organization; GONGO	Social mobilization
		Resettlement of affected people
		Command and coordination
		Information release
		Foreign affairs management
		Disaster assessment and report
		Prevent secondary disasters
		Maintenance of social security
Social service organization	Social group; Economic groups; Private non-enterprise units; State-owned enterprises	Medical treatment and epidemic prevention
		Resettlement of affected people
		Repair infrastructure
		Reconstruction
		Prevent secondary disasters
		Maintenance of social security
Civil society organizations	Community social organizations; Public welfare non-governmental organizations; Public Welfare Union;NGO;NPO	Social mobilization
		Search and rescue people
		Resettlement of affected people
		Repair infrastructure
		Reconstruction
		Maintenance of social security
Enterprise social organization	Private enterprises	Social mobilization
		Repair infrastructure
		Reconstruction
		Maintenance of social security

## References

[1] AghaKouchak A., Huning L.S., Chiang F., Sadegh M., Vahedifard F., Mazdiyasni O., Moftakhari H., Mallakpour I. (2018). How do natural hazards cascade to cause disasters?. Nature.

[2] Qiao S., Liu A.W., Yang J.S., Zhang D.N., Chen K., Chen X.L., Xu G.Y. Discussion on the Earthquake Disaster Chain and Cascading Effect. Proceedings of the 5th Annual Meeting of Risk Analysis Council of China Association for Disaster Prevention.

[3] Mascarello L.N., Quagliotti F. (2017). Challenges and Safety Aspects of a Disaster Relief Exercise. J. Intell. Robot. Syst..

[4] Borja A.M., Triantis K. (2007). A conceptual framework to evaluate performance of non-profit social service organisations. Int. J. Technol. Manag..

[5] Tierney K.J., Trianor J.E. (2003). Networks and Resilience in the World Trade Center Disaster.

[6] Moynihan D.P. (2009). The Network Governance of Crisis Response: Case Studies of Incident Command Systems. J. Public Adm. Res. Theory.

[7] Paul B.K. (2003). Relief assistance to 1998 flood victims: A comparison of the performance of the government and NGOs. Geogr. J..

[8] Chen J., Yao D.-Q., Liang L. (2017). Pre-positioning of relief inventories for non-profit organizations: A newsvendor approach. Ann. Oper. Res..

[9] Eikenberry A.M., Arroyave V., Copper T. (2007). Administrative Failure and the International NGO Response to Hurricane Katrina. Public Adm. Rev..

[10] Antronico L., Coscarelli R., De Pascale F., Condino F. (2019). Social Perception of Geo-Hydrological Risk in the Context of Urban Disaster Risk Reduction: A Comparison between Experts and Population in an Area of Southern Italy. Sustainability.

[11] UNHCR A Community-based Approach in UNHCR Operations. https://www.refworld.org/pdfid/47da54722.pdf.

[12] Skarbek E.C. (2014). The Chicago Fire of 1871: A bottom-up approach to disaster relief. Public Choice.

[13] Thaler T., Seebauer S. (2019). Bottom-up citizen initiatives in natural hazard management: Why they appear and what they can do?. Environ. Sci. Policy.

[14] Ali M.S.S., Arsyad M., Kamaluddin A., Busthanul N., Dirpan A. Community based disaster management: Indonesian experience. Proceedings of the 1st International Conference on Global Issue for Infrastructure, Environment and Socio-Economic Development.

[15] Forino G., Von Meding J., Brewer G. (2019). Community based initiatives to mainstream climate change adaptation into disaster risk reduction: Evidence from the Hunter Valley (Australia). Local Environ..

[16] Naim K. (2006). Interagency communication networks during Emergencies: Boundary spanners multiagency coordination. Am. Rev. Public Adm..

[17] Simo G., Bies A.L. (2007). The Role of Nonprofits in Disaster Response: An Expanded Model of Cross-Sector Collaboration. Public Adm. Rev..

[18] Caruson K., MacManus S. (2008). Disaster Vulnerabilities: How Strong a Push toward Regionalism and Intergovernmental Cooperation. Am. Rev. Public Adm..

[19] Boin A. (2009). Meeting the Challenges of Trans-boundary Crises: Building Blocks for Institutional Design. Contingencies Crisis Manag..

[20] Kapucu N., Demiroz F. (2011). Measuring Performance for Collaborative Public Management Using Network Analysis Methods and Tools. Public Perform. Manag. Rev..

[21] Cent J., Grodzińska-Jurczak M., Pietrzyk-Kaszyńska A. (2014). Emerging multilevel environmental governance—A case of public participation in Poland. J. Nat. Conserv..

[22] Sinuany-Stern Z., Sherman H.D. (2014). Operations research in the public sector and nonprofit organizations. Ann. Oper. Res..

[23] Gao H. (2019). Cross-Province State Aid and the Development of NGOs after the 2008 Sichuan Earthquake. China J..

[24] Maglajlic R.A. (2019). Organisation and delivery of social services in extreme events: Lessons from social work research on natural disasters. Int. Soc. Work.

[25] Mamula-Seadon L., McLean I. (2015). Response and early recovery following 4 September 2010 and 22 February 2011 Canterbury earthquakes: Societal resilience and the role of governance. Int. J. Disaster Risk Reduct..

[26] Khalili S., Harre M., Morley P. (2015). A temporal framework of social resilience indicators of communities to flood, case studies: Wagga wagga and Kempsey, NSW, Australia. Int. J. Disaster Risk Reduct..

[27] Chiang Y.C., Huang Y.C. (2016). Exploring social resilience: insights into climate change adaptation gaps from an estuarine region of Taiwan. J. Mar. Sci. Technol. Taiwan.

[28] Garcia D., Rime B. (2019). Collective Emotions and Social Resilience in the Digital Traces after a Terrorist Attack. Psychol. Sci..

[29] Kruse S., Abeling T., Deeming H., Fordham M., Forrester J., Jülich S., Karanci A.N., Kuhlicke C., Pelling M., Pedoth L. (2017). Conceptualizing community resilience to natural hazards—The emBRACE framework. Nat. Hazards Earth Syst. Sci. Discuss..

[30] Kim H., Marcouiller D.W., Woosnam K.M. (2018). Rescaling social dynamics in climate change: The implications of cumulative exposure, climate justice, and community resilience. Geoforum.

[31] Ludin S.M., Rohaizat M., Arbon P. (2019). The association between social cohesion and community disaster resilience: A cross-sectional study. Health Soc. Care Community.

[32] Zebardast E. (2013). Constructing a social vulnerability index to earthquake hazards using a hybrid factor analysis and analytic network process (F’ANP) model. Nat. Hazards.

[33] Werner T. (2015). Gaining Access by Doing Good: The Effect of Sociopolitical Reputation on Firm Participation in Public Policy Making. Manag. Sci..

[34] Malone E.L., Kinnear S. (2015). How and why: Complementary analyses of social network structures and cultural values: Improving flood response networks in Queensland, Australia. Qual. Quant..

[35] Hogg R.A., Varda D. (2016). Insights into Collaborative Networks of Nonprofit, Private, And Public Organizations That Address Complex Health Issues. Health Aff..

[36] Urrea G., Villa S., Goncalves P. (2016). Exploratory analyses of relief and development operations using social networks. Socio-Econ. Plan. Sci..

[37] Mukhtarov F., Dieperink C., Driessen P. (2018). The Influence of Information and Communication Technologies on Public Participation in Urban Water Governance: A Review of Place-based Research. Environ. Sci. Policy.

[38] Moroto H., Sakamoto M., Ahmed T. (2018). Possible factors influencing NGOs’ project locations for disaster management in Bangladesh. Int. J. Disaster Risk Reduct..

[39] Simsa R., Rameder P., Aghamanoukjan A., Totter M. (2019). Spontaneous Volunteering in Social Crises: Self-Organization and Coordination. Nonprofit Volunt. Sect. Q..

[40] Musial K., Bródka P., De Meo P. (2019). Analysis and Applications of Complex Social Networks 2018. Complexity.

[41] Li M.F., Zhao Y.X., He L.R. (2015). The parameter calibration and optimization of social force model for the real-life 2013 Ya’anearthquake evacuation in China. Saf. Sci..

[42] Aslam W., Butt W.H., Anwar M.W. A Systematic Review on Social Network Analysis—Tools, Algorithms and Frameworks. Proceedings of the International Conference on Computing and Big Data (ICCBD).

[43] The Central People’s Government of the People’s Republic of China. http://www.gov.cn/jrzg/2008-05/19/content_981852.htm.

[44] Tang J., Wang T. (2009). Efficient Social Network Approximate Analysis on Biosphere Based on Network Structure Characteristics, SNA-KDD09. The 3rd Workshop on Social Network Mining and Analysis.

[45] Lu R., Iqbal U., Li Y.-C. (2017). Two new computational methods for data analysis: A social network analysis-based classifier and the GEEORD SAS module. Comput. Methods Programs Biomed..

[46] Wasserman S., Faust K. (1994). Social Network Analysis: Methods and Applications.

[47] Stephen P.B., Martin G.E. (1993). Two algorithms for computing regular equivalence. Soc. Netw..

[48] Comfort L.K. (2002). Rethinking Security: Organizational Fragility in Extreme Events. Public Adm. Rev..

[49] Parker C.F., Stern E.K., Paglia E., Brown C. (2009). Preventable Catastrophe? The Hurricane Katrina Disaster Revisited. J. Contingencies Crisis Manag..

[50] Van der Vegt R.G. (2018). A literature review on the relationship between risk governance and public engagement in relation to complex environmental issues. J. Risk Res..

[51] Voss J.P., Amelung N. (2016). Innovating public participation methods: Technoscientization and reflexive engagement. Soc. Stud. Sci..

